# Biological Screening of *Glycyrrhiza glabra* L. from Different Origins for Antidiabetic and Anticancer Activity

**DOI:** 10.3390/ph16010007

**Published:** 2022-12-21

**Authors:** Rizwan Ahmad, Aljawharah Alqathama, Mohammed Aldholmi, Muhammad Riaz, Mohammed H. Mukhtar, Fatema Aljishi, Ebtihal Althomali, Muntathir Ali Alamer, Mohammed Alsulaiman, Abdulmalik Ayashy, Mohsen Alshowaiki

**Affiliations:** 1 Department of Natural Products and Alternative Medicine, College of Clinical Pharmacy, Imam Abdulrahman Bin Faisal University, Dammam 31441, Saudi Arabia; 2 Department of Pharmacognosy, Faculty of Pharmacy, Umm Al-Qura University, Makkah 21955, Saudi Arabia; 3 Department of Pharmacy, Shaheed Benazir Bhutto University, Sheringal 18050, Khyber Pakhtunkhwa, Pakistan; 4 Faculty of Medicine, Umm Al-Qura University, Makkah 21955, Saudi Arabia; 5 Prince Sultan Cardiac Center, Al Sulimaniyah 4th, Al Hofuf 36441, Saudi Arabia; 6 College of Clinical Pharmacy, Imam Abdulrahman Bin Faisal University, Dammam 34212, Saudi Arabia

**Keywords:** licorice, glucose uptake, glutathione peroxidase, antidiabetic, PCA

## Abstract

Background: Geographical variation may affect the phytochemistry as well as the biological activities of Glycyrrhiza glabra (licorice) root. Herein, a series of biological activities were performed to evaluate the impact of geographical origin on the biological potential of eight different licorice samples. Methodology: Cell culture studies were performed for cytotoxicity (MCF7, HCT116, HepG2, and MRC5), glucose uptake assay (HepG2), and glutathione peroxidase activity (HepG2), whereas α-amylase inhibition activity was tested for antidiabetic potential. Results: The Indian sample was observed to be more cytotoxic against MCF7 (22%) and HCT116 (43%) with an IC_50_ value of 56.10 (±2.38) μg/mL against the MCF7 cell line. The glucose uptake was seen with a mean value of 96 (±2.82) and a range of 92–101%. For glutathione peroxidase activity (GPx), the Syrian (0.31 ± 0.11) and Pakistani samples (0.21 ± 0.08) revealed a significant activity, whereas the Palestinian (70 ± 0.09) and Indian samples (68±0.06) effectively inhibited the α-amylase activity, with the lowest IC_50_ value (67.11 ± 0.97) μg/mL for the Palestinian sample. The statistical models of PCA (principal component analysis) and K-mean cluster analysis were performed to correlate the geographical origin, extract yield, and biological activities for the eight licorice samples of different origins. Conclusion: The licorice samples exhibited significant cytotoxic, GPx, and α-amylase inhibitory activity. The samples with higher extract yield showed more potential in these biological activities.

## 1. Introduction

*Glycyrrhiza glabra* is commonly known as licorice; it is considered the most important species of the *Glycyrrhiza* genus [1,2]. It is widely distributed globally, mostly in Asia, but it is also found in Europe, Africa, and Australia [3]. *Glycyrrhiza glabra* has considerable medicinal, pharmaceutical, and industrial value [4]. The roots of Glycyrrhiza glabra have been particularly well known for their medicinal effects since ancient times [5]. The populations of Rome, Greece, India, and China, use the roots to treat respiratory ailments such as asthma and bronchitis [5,6]. Consequently, pharmaceutical companies use *Glycyrrhiza glabra* in many cough syrup preparations [3]. Moreover, the strong sweet taste of the roots is of significant interest to industrial bodies. It acts as a sweetener in many tobacco products, chewing gums, ice cream flavors, and candies [3,7]. Notably, only some species are very sweet [7]. This sweet taste of *Glycyrrhiza glabra* is linked to the level of a triterpenoid saponin known as glycyrrhizin. It is considered the primary phytochemical and most abundant compound (10–25%) of the *Glycyrrhiza glabra* root extract [1,7]. Additionally, it has been used as a quality marker in the pharmacopeias of countries such as Japan and China [8,9]. In the plant, it is present as glycyrrhizic acid salt [10]. In several studies, geographical, environmental, and genetic factors were found to influence phytochemical levels, including that of glycyrrhizic acid [1]. Zhang et al. tested the glycyrrhizic acid content of five samples from different locations in China and found significant glycyrrhizic acid variations between the samples. The soil components were proposed as the most crucial factor in the abovementioned variation [11]. Similarly, the climate temperature influenced the glycyrrhizic acid levels of various Iranian *Glycyrrhiza glabra* roots from many areas in samples investigated by Hosseini et al. [12]. A substantial amount of literature has been published on the *Glycyrrhiza glabra* extracts and their phytochemical cytotoxic activities. For example, the ethanolic root extract of *Glycyrrhiza glabra* suppresses the growth of the (MDA-MB-231) breast cancer cell line in a dose-dependent manner [13]. Equally, the proliferation of the (T47D, MCF7, MDA-MB-231, and MDA-MB-361) breast cancer cell lines, the SiHa cervical cancer cell line, and the (A2780) ovarian cancer cell line was notably inhibited by the ethyl acetate root extract. A considerable amount of 18-β glycyrrhetic acid was found when a phytochemical analysis was performed [14]. The compound showed anticancer activity against A549 lung cancer cells when examined by Luo et al. [15]. Additionally, glycyrrhizic acid was also found to be cytotoxic in numerous studies. For instance, it showed significant antileukemic activity in K562 chronic myeloid leukemia cells and EL-4 lymphoma-bearing mice [16]. Furthermore, it induced apoptotic cell death in gastric cancerous cells [17]. Similar results were found in a study by Cai et al., when hepatic cancer cells were used [18]. The exact mechanism of action is unclear, but several mechanisms have been proposed for this anticancer activity [3]. Other biological activities such as anti-inflammatory, antioxidant, antimicrobial, and antiviral have been proven for *Glycyrrhiza glabra* extracts or its derivatives in numerous studies [1,19,20,21,22]. However, few studies have discussed the antidiabetic effects. For example, Takii et al. studied the effect of glycyrrhizin administration on non-insulin-dependent diabetic rats. After eight weeks, the blood glucose levels were suppressed remarkably in the treated rats compared to controlled non-insulin-dependent diabetic rats (*p* < 0.05). Likewise, the postprandial glucose level dropped notably after 60 min of glucose administration [23]. Similar findings were obtained by Sen et al. when the streptozotocin-induced diabetic model was used [24]. Glabridin could be suggested as the chemical constituent responsible for antidiabetic activity [25]. 

To the best of our knowledge, there are no studies on the correlation between the quality and origin of *Glycyrrhiza glabra* and its cytotoxic and antidiabetic activities. Thus, we attempt to investigate that correlation by using glycyrrhizic acid level as a quality marker.

## 2. Results

**Figure 1:**
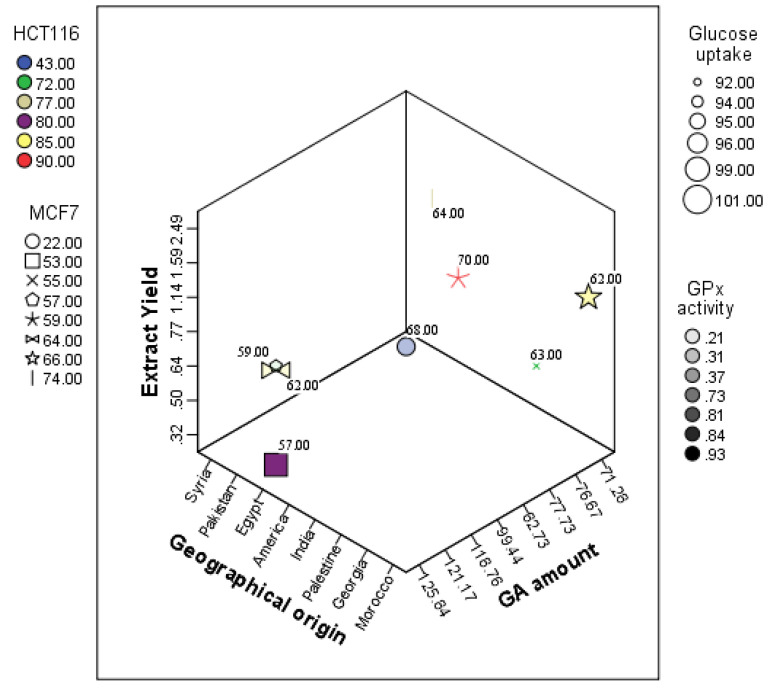
Three-dimensional representation of the licorice extract yield and GA amount vs. activities.

For descriptive analysis, principal component analysis, and K-mean cluster analysis, SPSS (Statistical Package for the Social Sciences) Version 22.0 was used for data entry and correlation analysis. A multi-view (three-dimensional) representation of the extract yield and GA amount versus the pharmacological activities (cytotoxicity, GPx, and glucose uptake assay) is presented in Figure 1.

### 2.1. Descriptive Analysis

#### 2.1.1. Cytotoxicity Assay

The cytotoxicity for these samples was observed with a mean value of 56.25 (±15.41) for MCF7 and 75.50 (±14.60) for HCT116, whereas the ranges (%) observed were 22–74 and 43–90 for MCF7 and HCT116, respectively. The lowest % viability for both cell lines was observed in the Indian-origin sample, i.e., 22% (MCF7) and 43% (HCT116), as shown in Table 1. This sample, upon further MTT assay investigation (six different concentrations for IC_50_) in MRC5, MCF7, and HCT116 cell lines revealed the lowest IC_50_ value of 56.10 (±2.38) μg/mL against the MCF7 cell line, as shown in Table 2. 

#### 2.1.2. The Effect on Glucose Uptake

A mean value of 96 (±2.82) with a range (%) of 92.00–101.00 was observed for the glucose utilization assay (HepG2) in these eight different geographical samples. The glucose uptake assay revealed the lack of any significant (*p* < 0.05) increase in glucose uptake and its utilization in HepG2 cells (100 μg/mL), as shown in Table 1. The standard drug Metformin showed a value of 118% (±0.07). 

#### 2.1.3. The Effect on Glutathione Peroxidase Activity (GPx)

The GPx activity (100 μg/mL) revealed a mean value of 0.64 (±0.29) within the range of 0.21–0.93. The study revealed a significant GPx activity for the Syrian (0.31 ± 0.11; *p* = 0.01) and Pakistani samples (0.21 ± 0.08; *p* = 0.01) as compared to the standard drug Quercetin (0.36 ± 0.10; *p* = 0.01). The GPx activity results for all the samples are shown in Table 1.

#### 2.1.4. The Effect of Extracts on α-Amylase Activity

The α-amylase activity was observed with a mean of 63.12 (±0.29) and a range (%) of 0.21–0.93. The initial screening for these samples (500 μg/mL) exhibited a significant inhibition for α-amylase (>50%); hence, they were further investigated at six different concentrations in order to determine the IC_50_ value. The samples observed with the highest inhibition during initial screening were the Palestinian (70 ± 0.09) and Indian samples (68 ± 0.06), as shown in Table 1. For IC_50_ value determination, the lowest value was observed for the Palestinian (67.11 ± 0.97), followed by the Indian sample (74.87 ± 1.26) μg/mL. The IC_50_ values observed for all the samples are reported in Table 3. 

### 2.2. Principal Component Analysis

**Figure 2:**
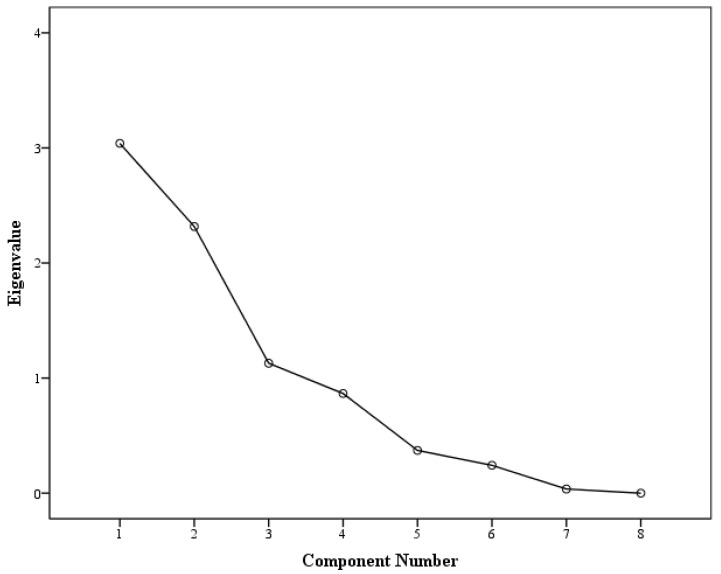
Scree plot for the component analysis of *Glycyrrhiza glabra* activities.

**Figure 3:**
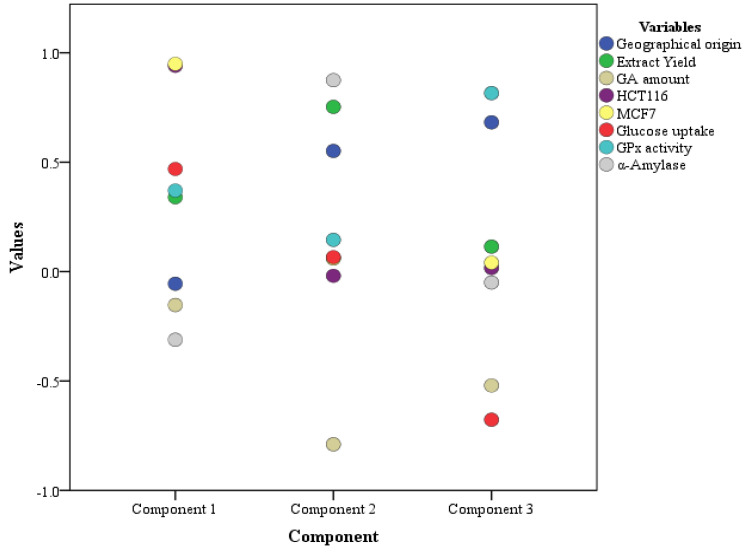
Distribution of data variables in components with expected values.

The PCA data for *Glycyrrhiza glabra* suggested three components, as shown in the scree plot Figure 2 and Table 4. The three components showed an individual and cumulative %variability of PC1 29.774 (29.774), PC2 28.607 (58.381), and PC3 23.467 (81.847). PC1, with more %variability, was loaded with cytotoxicity activities (HCT116 and MCF7) only. This reveals a high %variation of cytotoxic activities in relation to other biological activities, as well as a lack of correlation with the geographical origin, extract yield, and GA amount in these samples. As shown in Table 1, all the geographical origin samples exhibited cytotoxic potential with a similar pattern; however, the Indian and Georgian samples were significantly more cytotoxic. Yet, none of these samples had a high extract yield or GA amount either. PC2, with a variability of 28.607%, was loaded with the geographical origin, extract yield, and GA amount along with the α-amylase activity. This shows α-amylase activity to be more correlated with extract yield and GA amount, as all the *Glycyrrhiza glabra* samples of different geographical origins exhibited significant α-amylase activity. The last component, i.e., PC3 with a variability of 23.467%, was loaded with the geographical origin, glucose uptake, as well as GPx activity. PC3 suggests a higher correlation between glucose uptake and GPx activity, and geographical origin. For glucose uptake, more activity was shown by the geographical samples with either a high extract yield (Morocco, 99 ± 0.05; Palestine, 96 ± 0.09) or GA amount (Syria, 101 ± 0.07; Egypt, 96 ± 0.07). Likewise, GPx activity was observed to be more significant in the Pakistani and Syrian samples (0.21 ± 0.08 and 0.31 ± 0.11) with a high GA amount (*p* = 0.01). A graphical representation is also shown in Figure 3. 

### 2.3. K-Mean Cluster Analysis

**Figure 4:**
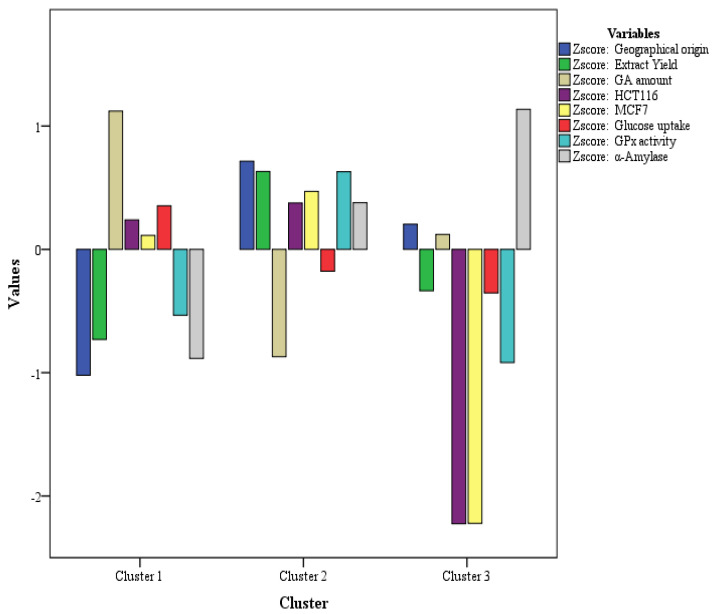
Data distribution in various K-clusters with values.

The K-mean suggested three clusters for *Glycyrrhiza glabra* samples, where all the samples were classified on the basis of extract yield and GA amount vs. activities. The three clusters consist of cluster (samples) 1(3), 2(4), and 3(1). Cluster one consisted of three samples with a higher GA amount reported (Syria, Pakistan, and Egypt), whereas cluster 2 consisted of four samples with a higher amount of extract yield (America, Palestine, Georgia, and Morocco). Figure 4 represents the cluster numbers/geographical origin samples with the corresponding activities exhibited by these samples. As evident, cluster 1, i.e., with a higher amount of GA, showed the highest glucose uptake activity, with a minimal activity potential for cytotoxicity (HCT116, MCF7). Cluster 2, i.e., with higher extract yield samples, exhibited almost all the activities except glucose uptake, whereas cluster 3 (one sample only i.e., Indian origin) exhibited a high potential for α-amylase activity. The K-mean cluster further supports the data from PCA analysis, where the GA amount was observed with a negative correlation for most activities. Hence, it may be concluded that the *Glycyrrhiza glabra* extracts from various geographical origins contain numerous phytochemicals which play an important role in different activities. The presence of GA in high amounts is not a guarantee that a sample will exhibit all the biological activities. The cluster distributions with the samples, F-values, and *p*-values are shown in Table 5. 

## 3. Discussion

This is the first study to report the phytochemical profile and evaluate the quality of *Glycyrrhiza glabra* samples of different geographical origins using various biological activities. The phytochemical quantification for GA amount was conducted in our previously published study [26], and the extracts thus quantified were subjected to multiple biological assays in order to correlate the *Glycyrrhiza glabra* phytochemistry with its biological potential. Eight *Glycyrrhiza glabra* samples of different origins were found and collected from Al-Khobar, an eastern province of Saudi Arabia. The green extraction and quantification were followed by a set of biological activities, including cytotoxicity, GPx, glucose uptake, and α-amylase activity. Statistical models were used to establish the correlation between the extract yield, GA amount, and biological activities. 

The initial screening for cytotoxicity (100 μg/mL) revealed a significant potential against HCT116 and MCF7, especially in the samples with a higher GA amount (Syria, Pakistan, and Egypt). However, the sample of Indian origin demonstrated a significantly higher cytotoxic effect against the HCT-116 and MCF7 cell lines. In order to determine the IC_50_ value for cytotoxicity, the Indian sample was studied further against the HCT116, MCF7, and MRC5 cell lines with different dose ranges (Table 2). In the context of GA amount, the Indian sample showed a lower amount as compared to other samples, which suggests that the cytotoxic effects may not be linked to GA content only. A number of other chemical constituents in the extract may play a vital role in cytotoxic and anticancer effects, including licochalcone-A [27,28,29,30] glycyrrhizinic acid [31], 18b-glycyrrhetinic acid [31,32,33,34,35,36], Isoliquiritigenin [37], formononetin [38], and glabridin [39]. 18b-glycyrrhetinic acid, one of the derivatives of glycyrrhetinic acid, is present in a higher concentration in the *Glycyrrhiza glabra* root as compared to 18α-glycyrrhetinic acid [40,41], being reported with a dose-dependent cytotoxic effect against the colon cancer cell line HCT-116 [32]. Likewise, glycyrrhetinic acid and glycyrrhizic acid have been reported to induce apoptosis in cancer cells [31,42,43] with an established cytotoxic potential of *Glycyrrhiza glabra* root, as previously reported [44,45,46].

Glucose uptake activity is essential for studying the metabolic diseases of cancer, diabetes, myocardial infarction, etc. [47]. A number of pathways are involved in the regulation of glucose in cancer cells, posing hurdles in the pathway of PI3K signaling or the selective blockage of glucose transporter type1 (GLUT1), thereby resulting in low glucose uptake [48,49]. Most of the available drugs are known to exert the anticancer effect via targeting GLUT1 to deprive tumor cells of glucose [48,50]. Herein, although the licorice samples of Moroccan and Syrian origin revealed a high glucose uptake, the comparative activity was lower than for the standard drug metformin. A dose-dependent stimulation of glucose uptake has been reported for licorice constituents such as glabridin [25] and glycyrrhizin [51]. 

Glutathione peroxidase (GPx) plays an important role in protection from oxidative damage, where it converts reduced glutathione to oxidized glutathione. An abnormal level of GPx has been reported in free radical-related disorders [52,53]. In our study, liver cell line lysates revealed a significant GPx activity for the Syrian, Pakistani, and Indian-origin samples as compared to the standard drug used. To the best of our knowledge, this assay is performed for the first time using *Glycyrrhiza glabra* extracts of different geographical origins. GPx is known for its complex effect on the development and progression of cancer due to its vital role in modulating the intracellular ROS [54]. An imbalance in essential ROS may lead to lessened cell growth with the encouragement of apoptotic pathways, whereas a surplus of cellular ROS plays a vital role in facilitating the multifaceted cascade of events leading to cell death via apoptotic pathways. For GPx, its high level may prevent oxidative damage and inflammation, although it may also block apoptotic cell death, hence leading to a higher survival rate for altered cells. This indicates the complex role of GPx, i.e., excess of GPx may allow for tumor survival and growth that may be reduced by the intake of suitable natural alternatives, such as licorice. Several in vivo toxicity studies already confirmed the role of licorice in normalizing or restoring the GPx level in the toxicity studies models [55,56,57,58]. The inhibition of carbohydrate digesting enzyme, i.e., α-amylase, is one of the investigative targets for treating diabetes [59]. The *Glycyrrhiza glabra* samples from Palestine and India revealed a significant α-amylase inhibitory activity as compared to the standard drug acarbose. Previous studies have reported a more effective inhibition of α-amylase than acarbose [60], which is in line with our current results.

The statistical models of PCA and K-mean cluster analysis of correlation revealed more activities for the licorice samples with higher extract yields. The samples with higher amounts of GA were found to have significant glucose uptake activity only, whereas the remaining tested activities were found to be significantly better in the samples with higher extract yields. This may be due to the presence of various other chemical classes present in these extracts, which imparts potential to these samples as observed in the cytotoxic, GPx, and α-amylase activities. Hence, it may be concluded that the presence of one chemical class or constituent may not be claimed for all the biological or pharmacological activities. The presence of multiple chemical constituents or phytochemical classes may also result in a synergistic effect, hence showing comparatively more significant results when tested in various throughput screenings.

## 4. Materials and Methods

### 4.1. Extraction and Phytochemical Analysis

The *Glycyrrhiza glabra* samples of different geographical origins (Syria, Egypt, America, Pakistan, India, Palestine, Georgia, and Morocco) were extracted with the help of the ultrasonication technique using water as a green solvent. The green analytical method of UHPLC-MS/MS was applied to quantify the amount of glycyrrhizic acid (GA) in each sample. The details regarding the optimization and validation for green extraction and analysis of GA in these samples were reported in our previous study [26]. For convenience, the extract yield and GA amount/sample are reported in Table 1 herein.

### 4.2. Cell Culture Cytotoxicity Studies

Four cancer cell lines, MCF7 (human breast adenocarcinoma, ATCC-HTB22), HCT116 (human colorectal carcinoma, ATCC-CCL247), HepG2 (hepatocellular carcinoma, ATCC HB-8065), and one normal fibroblast cell line, MRC5 (normal human fetal lung fibroblast, ATCC-CCL171), were used in this study. Three of the cell lines (MCF7, HCT116, and MRC5) were maintained in Roswell Park Memorial Institute Medium (RPMI-1640, Gibco, Life Technologies, Carlsbad, CA, USA), while the HepG2 cell line was cultured in Dulbecco’s modified Eagle’s medium (DMEM, Gibco, Life Technologies, Carlsbad, CA, USA). All of the cell lines were maintained at 37 °C in 5% CO_2_ and 100% relative humidity. All media were supplemented with 10% heat-inactivated fetal bovine serum (FBS, Gibco) and 1% penicillin–streptomycin antibiotic, consisting of 10,000 units of penicillin and 10,000 µg of streptomycin (Gibco) per mL.

#### Determination of Cytotoxicity and Selectivity

The cytotoxicity of the extracts was evaluated by MTT assay, as previously reported [61]. The MCF7, HCT116, and MRC5 cell lines were tested to determine the cytotoxicity and selectivity of the extracts, while HepG2 was used to determine the effective concentrations of the extracts. Concentrations that did not significantly influence cell viability were further investigated in other assays. The MCF7 and HCT116 cells were cultured separately in 96-well plates (3 × 10^3^/well) and incubated at 37 °C overnight. The first set of experiments screened the samples for cytotoxic activity at 100 μg/mL concentration (DMSO 0.4%; *n* = 3). Plates were incubated for 48 h, after which MTT was added to each well and the plates were incubated further for 3 h. The supernatant was removed, and the MTT product was solubilized by adding DMSO to each well. Absorbance was read using a multi-plate reader (BIORAD, PR 4100, Hercules, CA, USA). The optical density of the purple formazan A550 was proportional to the number of viable cells, which was calculated as an inhibition percentage compared to control cells and listed in Table 1. The extracts with the highest percentage of inhibition were selected for the second set of experiments to determine the half-maximal inhibitory concentration (IC_50_) and selectivity using the same cancer cell lines and the same fibroblast cell line. The range of extract concentrations tested were 500, 250, 100, 50, 10, and 1 μg/mL, and doxorubicin was used as a positive control to determine the IC_50_ values using GraphPad Prism (San Diego, CA, USA) (Table 2).

### 4.3. Glucose Uptake Assay

Glucose uptake was evaluated for the *Glycyrrhiza glabra* extracts as previously outlined by Odeyemi et al. [62]. After seeding HepG2 cells (5000 cells/well) in a 96-well plate and growing them overnight in a 37 °C humidified incubator with 5% CO_2_, cells were exposed to extract concentrations that had no influence on cell viability and incubated at 37 °C for 48 h, with 0.2 µg/mL metformin as a positive control. The culture medium was then aspirated, and the cells were incubated with an incubation medium containing DMEM diluted with 8 mM glucose, 0.1% bovine serum albumin (BSA), and PBS for 3 h at 37 °C. The assay was carried out by transferring the incubation medium to a new 96-well plate to measure the glucose concentration in the medium, following the manufacturer’s protocol (GAGO20, Sigma Aldrich, St Louis, MO, USA). Absorbance was measured at 550 nm on a multi-plate reader (BIORAD, PR 4100, Hercules, CA, USA). We calculated the amount of glucose used by the cells by subtracting the cell-containing wells from the cell-free wells.

### 4.4. Determination of Glutathione Peroxidase Activity

The activity of glutathione peroxidase (GPx) was evaluated using HepG2 cell lysates in a colorimetric assay, performed according to the manufacturer’s protocol (ab102530, Abcam, Cambridge, UK). HepG2 cells (5 × 10^5^ cells/ well) were seeded in a 12-well plate and treated with extract concentrations that do not influence cell viability. These were incubated for 48 h, with 25 µg/mL of quercetin used as a positive control, after which the cells were collected and washed with cold PBS. The collected cells were depleted of all GSSG by incubating the samples with glutathione reductase (GR) and reduced glutathione (GSH) for 15 min. GPx activity was determined by adding cumene hydroperoxide and incubating for 5 min. The absorbance was measured before and after adding cumene hydroperoxide at 340 nm, using a multi-plate reader (BIORAD, PR 4100, Hercules, CA, USA). The decrease in NADPH (measured at OD = 340 nm) is proportional to GPx activity. The concentration of GPx in the test samples was calculated as recommended by the manufacturer’s protocol.

### 4.5. α-Amylase Inhibition Activity

All extracts were initially tested at 500 μg/mL, and those that demonstrated an inhibitory effect on the α-amylase enzyme were further investigated at a range of concentrations (1000, 500, 100, 50, 25, and 10 μg/mL), with acarbose as a positive control. The inhibitory activity of α-amylase was ascertained as described by Quan et al. [63]. An aliquot of 1 mg α-amylase from *Aspergillus oryzae* in a phosphate buffer was prepared, and 20 μL was added to each well in a 96-well plate along with 20 μL of the extract samples that were diluted in a phosphate buffer. After mixing, the plate was incubated for 10 min at 37 °C, then 30 μL of starch (0.05% in deionized water) was added to each reaction well and incubated for a further 8 min at 37 °C. The reaction was then halted by the addition of 20 μL of hydrochloric acid (1M) followed by 100 μL of iodine reagent (0.25 mM) to each well. Control wells were set up by replacing the enzyme with buffer and adding acarbose to create a positive control. Absorbance was measured using a multi-plate reader (BIORAD, PR 4100, Hercules, CA, USA) at 550 nm for each well, calculating the percentage of inhibition utilizing the equation:

% inhibition = (A−C/B−C) × 100,

where A = the absorbance of the reaction mixture in the presence of the extract, B = the absorbance of the mixture without the enzyme, and C = the absorbance of the reaction mixture in the absence of any extract.

### 4.6. Statistical Analysis

The results are expressed as the mean ± standard deviation (SD) from at least three independent experiments. All data were analyzed with a *p*-value of < 0.05 considered as significant in order to find the statistical significance between treated groups and controls using GraphPad Prism 9.2.0 (GraphPad, San Diego, CA, USA).

## 5. Conclusions

The tested *Glycyrrhiza glabra* samples revealed a significant potential in different biological activities, including cytotoxicity, glucose uptake, GPx, and α-amylase activity. The samples were effectively classified into groups consisting of higher extract yield and higher GA amount samples. The statistical analysis established a higher correlation for samples with higher extract yield vs. biological activities, as compared to the samples with a higher GA amount. Hence, it is of utmost importance to further investigate and explore the complete metabolomics profile along with in vitro and in vivo studies for all these *Glycyrrhiza glabra* samples in order to reveal the presence of phytochemical classes. This may provide a more effective data source for a clear demarcation of the quality of *Glycyrrhiza glabra* samples based on geographical origin, which is sensitive to various environmental, agricultural, storage, and transport factors.

## Figures and Tables

**Table 1:** Extract yield, GA amount, and the data for activity screening of licorice samples. (** *p* = 0.05, *** *p* = 0.01).

Geographical Origin	Extract Yield (g/10 g)	GA Amount (mg/10 g)	HCT116	MCF7	Glucose Uptake	GPx Activity	α-Amylase
Syria	0.5	118.76	85 ± 0.15	64 ± 0.12	101 ± 0.07	0.31 ± 0.11 ***	62 ± 0.07
Egypt	0.32	125.84	80 ± 0.18	53 ± 0.01	96 ± 0.07	0.93 ± 0.13	57 ± 0.14
America	2.49	77.73	77 ± 0.13	74 ± 0.15	95 ± 0.11	0.81 ± 0.25	64 ± 0.10
Pakistan	0.64	121.17	72 ± 0.22	57 ± 0.03	94 ± 0.09	0.21 ± 0.08 ***	59 ± 0.05
India	0.77	99.44	43 ± 0.21	22 ± 0.02	95 ± 0.09	0.37 ± 0.18 **	68 ± 0.06
Palestine	1.59	82.73	90 ± 0.15	59 ± 0.03	96 ± 0.09	0.84 ± 0.15	70 ± 0.09
Georgia	0.64	76.67	72 ± 0.17	55 ± 0.07	92 ± 0.12	0.93 ± 0.12	63 ± 0.07
Morocco	1.14	71.28	85 ± 0.21	66 ± 0.01	99 ± 0.05	0.73 ± 0.30	62 ± 0.13
Standards			Metformin		118 ± 0.08 **		
			Quercetin			1.59 ± 0.21 ***	
			Acarbose				78.41 ± 0.67

**Table 2:** Cytotoxicity and selectivity of the selected extract (MTT 48 h, IC_50_ ±SD μg/mL).

Geographical Origin	HCT116	MCF7	MRC5
India	100.3 ± 1.00	56.10 ± 2.38	91.00 ± 1.39
**Doxorubicin**	4.19 ± 1.23	3.11 ± 1.34	6.90 ± 0.95

**Table 3:** α-Amylase inhibitory activity of the selected extracts (IC_50_ ± SD μg/mL).

Country	IC_50_
Syria	93.46 ± 3.77
Egypt	120.6 ± 2.33
America	87.21 ± 2.73
Pakistan	110.6 ± 1.21
India	74.87 ± 1.26
Palestine	67.11 ± 0.97
Georgia	98.50 ± 1.58
Morocco	93.73 ± 1.09
Acarbose	80.86 ± 0.58

**Table 4:** The principal component analysis for licorice samples.

Components	PC1	PC2	PC3
Geographical origin	**−**0.056	**0.551**	**0.682**
Extract yield	0.340	**0.753**	0.114
GA amount	**−**0.153	**−0.789**	**−**0.520
HCT116	**0.940**	**−**0.019	0.017
MCF7	**0.950**	0.060	0.041
Glucose uptake	0.469	0.066	**−0.677**
GPx activity	0.370	0.145	**0.816**
α-amylase	**−**0.312	**0.875**	**−**0.050
**Individual %variance**	**29.774**	**28.607**	**23.467**
**Cumulative %variance**	**29.774**	**58.381**	**81.847**

**Table 5:** K-mean analysis for different geographical samples of licorice.

Factors	F-Value	Significance	Clusters	Samples
score: Geographical origin	7.267	0.033	1	3
Zscore: Extract yield	2.242	0.202	2	4
Zscore: GA amount	93.749	0.000	3	1
Zscore: HCT116	10.839	0.015	**Total**	**8**
Zscore: MCF7	12.846	0.011		
Zscore: Glucose uptake	0.245	0.792		
Zscore: GPx activity	2.211	0.205		
Zscore: α-amylase	3.766	0.101		

## Data Availability

Any data generated during this study is presented in this document.

## Abbreviations

MCF7 (human breast adenocarcinoma), HCT116 (human colorectal carcinoma), HepG2 (hepatocellular carcinoma), MRC5 (normal human fetal lung fibroblast), GPx (glutathione peroxidase), MDA-MB (M.D. Anderson—Metastatic Breast cells), SD (standard deviation), PCA (principal component analysis), GA (glycyrrhizic acid), ATCC (American Type Culture Collection), MTT (3-(4,5-dimethylthiazol-2-yl)-2,5-diphenyl-2H-tetrazolium bromide), DMSO (dimethyl sulfoxide), CO_2_ (carbon dioxide), PBS (phosphate-buffered saline), NADPH (nicotinamide adenine dinucleotide phosphate), OD (optical density).

## References

[1] Pastorino G., Cornara L., Rodrigues F., Beatriz P.P.O. (2018). Liquorice (*Glycyrrhiza glabra*): A phytochemical and pharmacological review. Phytother. Res..

[2] Sharifi-Rad J., Quispe C., Herrera-Bravo J., Herrera Belén L., Kaur R., Kregiel D., Uprety Y., Beyatli A., Yeskaliyeva B., Kırkın C. (2021). *Glycyrrhiza Genus*: Enlightening Phytochemical Components for Pharmacological and Health-Promoting Abilities. Oxidative Med. Cell. Longev..

[3] Kew S. *Glycyrrhiza glabra* L. Kew Science. https://powo.science.kew.org/taxon/urn:lsid:ipni.org:names:496941-1.

[4] Wahab S., Annadurai S., Abullais S.S., Das G., Ahmad W., Ahmad F., Kandasamy G., Vasudevan R., Ali S., Amir M. (2021). *Glycyrrhiza glabra* (Licorice): A comprehensive review on its phytochemistry, biological activities, clinical evidence and toxicology. Plants.

[5] Armanini D., Fiore C., Mattarello M.J., Bielenberg J., Palermo M. (2002). History of the endocrine effects of licorice. Exp. Clin. Endocrinol. Diabetes.

[6] Hasan M.K., Ara I., Mondal M.S.A., Kabir Y. (2021). Phytochemistry, pharmacological activity, and potential health benefits of *Glycyrrhiza glabra*. Heliyon.

[7] Isbrucker R.A., Burdock G.A. (2006). Risk and safety assessment on the consumption of Licorice root (*Glycyrrhiza* sp.), its extract and powder as a food ingredient, with emphasis on the pharmacology and toxicology of glycyrrhizin. Regul. Toxicol. Pharmacol..

[8] Ishimi Y., Takebayashi J., Tousen Y., Yamauchi J., Fuchino H., Kawano T., Inui T., Yoshimatsu K., Kawahara N. (2019). Quality evaluation of health foods containing licorice in the Japanese Market. Toxicol. Rep..

[9] Zhang Y., Wang C., Yang F., Sun G. (2019). A strategy for qualitative and quantitative profiling of glycyrrhiza extract and discovery of potential markers by fingerprint-activity relationship modeling. Sci. Rep..

[10] Dastagir G., Rizvi M.A. (2016). *Glycyrrhiza glabra* L. (Liquorice). Pak. J. Pharm. Sci..

[11] Zhang J.T., Xu B., Li M. (2011). Relationships between the bioactive compound content and environmental variables in *Glycyrrhiza uralensis* populations in different habitats of North China. Phyton-Int. J. Exp. Bot..

[12] Hosseini S.M.A., Souri M.K., Farhadi N., Moghadam M., Omidbaigi R. (2014). Changes in Glycyrrhizin Content of Iranian licorice (*Glycyrrhiza glabra* L.) Affected by Different Root Diameter and Ecological Conditions. Agric. Commun..

[13] Hamad G., Elaziz A., Hassan S., Shalaby M., Mohdaly A. (2020). Chemical Composition, Antioxidant, Antimicrobial and Anticancer Activities of Licorice (*Glycyrrhiza glabra* L.) Root and Its Application in Functional Yoghurt. J. Food Nutr. Res..

[14] Vlaisavljević S., Šibul F., Sinka I., Zupko I., Ocsovszki I., Jovanović-Šanta S. (2018). Chemical composition, antioxidant and anticancer activity of licorice from Fruska Gora locality. Ind. Crops Prod..

[15] Luo Y.H., Wang C., Xu W.T., Zhang Y., Zhang T., Xue H., Li Y.N., Fu Z.R., Wang Y., Jin C.H. (2021). 18β-Glycyrrhetinic Acid Has Anti-Cancer Effects via Inducing Apoptosis and G2/M Cell Cycle Arrest, and Inhibiting Migration of A549 Lung Cancer Cells. OncoTargets Therapy.

[16] Hostetler B.J., Uchakina O.N., Ban H., Mckallip R.J. (2017). Treatment of hematological malignancies with glycyrrhizic acid. Anticancer Res..

[17] Wang H., Ge X., Qu H., Wang N., Zhou J., Xu J., Zhou Y., Shi L., Qin Z., Jian Z. (2020). Glycyrrhizic Acid Inhibits Proliferation of Gastric Cancer Cells by Inducing Cell Cycle Arrest and Apoptosis. Cancer Manag. Res..

[18] Cai S., Zhun B., Bai Y., Zhang H., Zhai D., Xiao C., Tang Y., Yang L., Zhang X., Li K. (2020). Glycyrrhizic Acid-Induced Differentiation Repressed Stemness in Hepatocellular Carcinoma by Targeting c-Jun N-Terminal Kinase 1. Front. Oncol..

[19] Dos Leite C.S., Bonafé G.A., Carvalho Santos J., Real Martinez C.A., Marques Ortega M., Lima Ribeiro R. (2022). The Anti-Inflammatory Properties of Licorice (*Glycyrrhiza glabra*)-Derived Compounds in Intestinal Disorders. Int. J. Mol. Sci..

[20] Karahan F., Avsar C., Ozyigit I.I., Berber I. (2016). Antimicrobial and antioxidant activities of medicinal plant *Glycyrrhiza glabra* var. *glandulifera* from different habitats. Biotechnol. Biotechnol. Equip..

[21] Gupta V.K., Fatima A., Faridi U., Negib A.S., Shanker K., Rahuja K.N., Luqman S., Sisodia B.S., Saikia D., Darokaret M.P. (2008). Antimicrobial potential of *Glycyrrhiza glabra* roots. J. Ethnopharmacol..

[22] Huan C., Xu Y., Zheng W., Guo T., Pan H., Gao S. (2021). Research Progress on the Antiviral Activity of Glycyrrhizin and its Derivatives in Liquorice. Front. Pharmacol..

[23] Takii H., Kometani T., Nishimura T., Nakae T., Okada S., Fushiki T. (2001). Antidiabetic effect of glycyrrhizin in genetically diabetic KK-Ay mice. Biol. Pharm. Bull..

[24] Sen S., Roy M., Chakraborti A.S. (2011). Ameliorative effects of glycyrrhizin on streptozotocin-induced diabetes in rats. J. Pharm. Pharmacol..

[25] Sawada K., Yamashita Y., Zhang T., Nakagawa K., Ashida H. (2014). Glabridin induces glucose uptake via the AMP-activated protein kinase pathway in muscle cells. Mol. Cell. Endocrinol..

[26] Ahmad R., Aldholmi M., Alqathama A., Aldossary S., Bubshait S., Aljaber M., Abuhassan A., Aldarwish A., Alateeq L. (2021). Green and novel ultrasonic extraction with UHPLC-MSMS analysis of natural sweetener (Glycyrrhizic acid) from *Glycyrrhiza glabra*; a multifactorial mechanistic evaluation based on statistical analysis. Ultrason. Sonochemistry.

[27] Fu Y., Hsieh T.-c., Guo J., Kunicki J., Lee M.Y., Darzynkiewicz Z., Wu J.M. (2004). Licochalcone-A, a novel flavonoid isolated from licorice root (*Glycyrrhiza glabra*), causes G2 and late-G1 arrests in androgen-independent PC-3 prostate cancer cells. Biochem. Biophys. Res. Commun..

[28] Di Paola R.S., Zhang H., Lambert G.H., Meeker R., Licitra E., Rafi M.M., Zhu B.T., Spaulding H., Goodin S., Toledano M.B. (1998). Clinical and biologic activity of an estrogenic herbal combination (PC-SPES) in prostate cancer. N. Engl. J. Med..

[29] Rafi M.M., Rosen R.T., Vassil A., Ho C.-T., Zhang H., Ghai G., Lambert G., Di Paola R.S. (2000). Modulation of bcl-2 and cytotoxicity by licochalcone-A, a novel estrogenic flavonoid. Anticancer Res..

[30] Yo Y.-T., Shieh G.-S., Hsu K.-F., Wu C.-L., Shiau A.-L. (2009). Licorice and licochalcone-A induce autophagy in LNCaP prostate cancer cells by suppression of Bcl-2 expression and the mTOR pathway. J. Agric. Food Chem..

[31] Haghshenas V., Fakhari S., Mirzaie S., Rahmani M., Farhadifar F., Pirzadeh S., Jalili A. (2014). Glycyrrhetinic Acid inhibits cell growth and induces apoptosis in ovarian cancer a2780 cells. Adv. Pharm. Bull..

[32] Yuan H., Gao J. (2022). 18 beta-Glycyrrhetinic Acid Induces Reactive Oxygen Species-Mediated Apoptosis along with Cell Cycle Arrest in Colon Cancer Cells. Int. J. Pharmacol..

[33] Wang X.F., Zhou Q.-M., Lu Y.-Y., Zhang H., Huang S., Su S.-B. (2015). Glycyrrhetinic acid potently suppresses breast cancer invasion and metastasis by impairing the p38 MAPK-AP1 signaling axis. Expert Opin. Ther. Targets.

[34] Sharma G., Kar S., Palit S., Das P.K. (2012). 18β-glycyrrhetinic acid induces apoptosis through modulation of Akt/FOXO_3_a/Bim pathway in human breast cancer MCF-7 cells. J. Cell. Physiol..

[35] Pirzadeh S., Fakhari S., Jalili A., Mirzai S., Ghaderi B., Haghshenas V. (2014). Glycyrrhetinic acid induces apoptosis in leukemic HL60 cells through upregulating of CD95/CD178. Int. J. Mol. Cell. Med..

[36] Hibasami H., Iwase H., Yoshioka K., Takahashi H. (2006). Glycyrrhetic acid (a metabolic substance and aglycon of glycyrrhizin) induces apoptosis in human hepatoma, promyelotic leukemia and stomach cancer cells. Int. J. Mol. Med..

[37] Zheng H., Wang Y., Zhao H., Zhang J., Chai H., Tang T., Yue J., Guo A.M., Yang J. (2014). Downregulation of COX-2 and CYP 4A signaling by isoliquiritigenin inhibits human breast cancer metastasis through preventing anoikis resistance, migration and invasion. Toxicol. Appl. Pharmacol..

[38] Zhou R., Xu L., Ye M., Liao M., Du H., Chen H. (2014). Formononetin inhibits migration and invasion of MDA-MB-231 and 4T1 breast cancer cells by suppressing MMP-2 and MMP-9 through PI3K/AKT signaling pathways. Horm. Metab. Res..

[39] Hsieh M.J., Lin C.W., Yang S.F., Chen M.K., Chiou H.L. (2014). G labridin inhibits migration and invasion by transcriptional inhibition of matrix metalloproteinase 9 through modulation of NF-κ B and AP-1 activity in human liver cancer cells. Br. J. Pharmacol..

[40] Lee J.K., Kim J.H., Shin H.K. (2011). Therapeutic effects of the oriental herbal medicine Sho-saiko-to on liver cirrhosis and carcinoma. Hepatol. Res..

[41] Sabbioni C., Mandrioli R., Ferranti A., Bugamelli F., Saracino M.A., Forti G.C., Fanali S., Raggi M.A. (2005). Separation and analysis of glycyrrhizin, 18β-glycyrrhetic acid and 18α-glycyrrhetic acid in liquorice roots by means of capillary zone electrophoresis. J. Chromatogr. A.

[42] Aguirre D., Boya P., Bellet D., Faivre S., Troalen F., Benard J., Saulnier P., Hopkins-Donaldson S., Zangemeister-Wittke U., Kroemer G. (2004). Bcl-2 and CCND1/CDK4 expression levels predict the cellular effects of mTOR inhibitors in human ovarian carcinoma. Apoptosis.

[43] Mahdinejadiani K., Shirzad H., Fakhari S., Jalili A. (2015). An Evaluation the Effect of Glycyrrhetinic and Glycyrrhizic Acids Derived from Licorice Extract on Gastric Cancer Cell Lines. J. Babol Univ. Med. Sci..

[44] Rathi S., Suthar M., Patel P., Bhaskar V., Rajgor N. (2009). In-vitro cytotoxic screening of *Glycyrrhiza glabra* L. (Fabaceae): A natural anticancer drug. J. Young Pharm..

[45] Sheela M., Ramakrishna M., Salimath B.P. (2006). Angiogenic and proliferative effects of the cytokine VEGF in Ehrlich ascites tumor cells is inhibited by *Glycyrrhiza glabra*. Int. Immunopharmacol..

[46] Jo E.H., Kim S.-H., Ra J.-C., Kim S.-R., Cho S.-D., Jung J.-W., Yang S.-R., Park J.-S., Hwang J.-W., Aruoma O.I. (2005). Chemopreventive properties of the ethanol extract of chinese licorice (*Glycyrrhiza uralensis*) root: Induction of apoptosis and G1 cell cycle arrest in MCF-7 human breast cancer cells. Cancer Lett..

[47] Yamamoto N., Ueda-Wakagi M., Sato T., Kawasaki K., Sawada K., Kawabata K., Akagawa M., Ashida H. (2015). Measurement of glucose uptake in cultured cells. Curr. Protoc. Pharmacol..

[48] Vander Heiden M.G., Cantley L.C., Thompson C.B. (2009). Understanding the Warburg effect: The metabolic requirements of cell proliferation. Science.

[49] Engelman J.A., Chen L., Tan X., Crosby K., Guimaraes A.R., Upadhyay R., Maira M., McNamara K., Perera S.A., Song Y. (2008). Effective use of PI3K and MEK inhibitors to treat mutant Kras G12D and PIK3CA H1047R murine lung cancers. Nat. Med..

[50] Chan D.A., Sutphin P.D., Nguyen P., Turcotte S., Lai E.W., Banh A., Reynolds G.E., Chi J.-T., Wu J., Solow-Cordero D.E. (2011). Targeting GLUT1 and the Warburg effect in renal cell carcinoma by chemical synthetic lethality. Sci. Transl. Med..

[51] Sun Z., Tan Z., Peng C., Yi W. (2021). HK2 is associated with the Warburg effect and proliferation in liver cancer: Targets for effective therapy with glycyrrhizin Corrigendum in/10.3892/mmr. 2021.12143. Mol. Med. Rep..

[52] Kaynar H., Meral M., Turhan H., Keles M., Celik G., Akcay F. (2005). Glutathione peroxidase, glutathione-S-transferase, catalase, xanthine oxidase, Cu–Zn superoxide dismutase activities, total glutathione, nitric oxide, and malondialdehyde levels in erythrocytes of patients with small cell and non-small cell lung cancer. Cancer Lett..

[53] Jiao Y., Wang Y., Guo S., Wang G. (2017). Glutathione peroxidases as oncotargets. Oncotarget.

[54] Brigelius-Flohe R., Kipp A. (2009). Glutathione peroxidases in different stages of carcinogenesis. Biochim. Et. Biophys. Acta (BBA)-Gen. Subj..

[55] Muralidharan P., Balamurugan G., Babu V. (2009). Cerebroprotective effect of *Glycyrrhiza glabra* Linn. root extract on hypoxic rats. Bangladesh J. Pharmacol..

[56] Rajesh M., Latha M. (2004). Protective activity of *Glycyrrhiza glabra* Linn. on carbon tetrachloride-induced peroxidative damage. Indian J. Pharmacol..

[57] Zhang L., Yang Y., Yu L., Wang Y., Liu L., Fan X. (2011). Cardioprotective effects of *Glycyrrhiza uralensis* extract against doxorubicin-induced toxicity. Int. J. Toxicol..

[58] Huo H.Z., Wang B., Liang Y.K., Bao Y.Y., Gu Y. (2011). Hepatoprotective and antioxidant effects of licorice extract against CCl4-induced oxidative damage in rats. Int. J. Mol. Sci..

[59] Mccue P., Kwon Y.I., Shetty K. (2005). Anti-amylase, anti-glucosidase and anti-angiotensin i-converting enzyme potential of selected foods. J. Food Biochem..

[60] Fathima F., Rajeshkumar S. (2021). In Vitro Anti-Diabetic Activity of *Glycyrrhizaglabraethanolic* Extract. Ann. Rom. Soc. Cell Biol..

[61] Al-Salem H.S., Arifuzzaman M., Alkahtani H.M., Abdalla A.N., Issa I.S., Alqathama A., Albalawi F.S., Rahman A. (2020). A series of isatin-hydrazones with cytotoxic activity and cdk2 kinase inhibitory activity: A potential type ii atp competitive inhibitor. Molecules.

[62] Odeyemi S., Dewar J. (2020). In vitro antidiabetic activity affecting glucose uptake in hepg2 cells following their exposure to extracts of lauridia tetragona (lf) rh archer. Processes.

[63] Quan N.V., Tran H.-D., Xuan T.D., Ahmad A., Dat T.D., Khanh T.D., Teschke R. (2019). Momilactones a and b are α-amylase and α-glucosidase inhibitors. Molecules.

